# Supplementary material for: Health Information Seeking Experiences Among People with Disabilities: Results from the HINTS 2024
Source: medRxiv. 2025 Oct 15:2025.10.14.25338014. Preprint. [Version 1] doi: 10.1101/2025.10.14.25338014 (PMC12633093; doi:10.1101/2025.10.14.25338014)
Supplement: 1 [file NIHPP2025.10.14.25338014V1-supplement-1.pdf]

This is a pre-print and has not yet been peer-reviewed. This manuscript is under review at a peer-reviewed journal. Please cite the following for this article:

Velat M, James TG. Health information seeking experiences among people with disabilities: Results from the HINTS 2024. *medarxiv*.

Supplemental Table 1. Unweighted adjusted logistic regression results under Full Information Maximum Likelihood implemented in Mplus.

	Deaf	Blind	Mobility	Pain	Multiple
Seeking cancer information	**0.578 (0.415 to 0.806)**	**0.582 (0.419 to 0.810)**	0.763 (0.578 to 1.008)	0.852 (0.708 too 1.026)	**0.820 (0.704 to 0.955)**
Effort	1.230 (0.731 to 2.070)	**1.907 (1.160 to 3.136)**	1.374 (0.917 to 2.058)	**1.412 (1.092 to 1.825)**	**1.628 (1.324 to 2.002)**
Frustration	1.418 (0.848 to 2.371)	1.611 (0.980 to 2.647)	1.242 (0.819 to 1.884)	**1.522 (1.175 to 1.972)**	**1.639 (1.328 to 2.021)**
Understand	1.246 (0.740 to 2.098)	**1.763 (1.071 to 2.901)**	**1.646 (1.092 to 2.479)**	1.118 (0.861 to 1.451)	**1.445 (1.173 to 1.781)**
Quality	0.966 (0.582 to 1.603)	**1.781 (1.038 to 3.057)**	0.946 (0.629 to 1.422)	1.278 (0.983 to 1.660)	**1.363 (1.102 to 1.686)**

medRxiv preprint doi: https://doi.org/10.1101/2025.10.14.25338014; this version posted October 15, 2025. The copyright holder for this preprint **(which was not certified by peer review)** is the author/funder, who has granted medRxiv a license to display the preprint in perpetuity.
It is made available under a CC-BY 4.0 International license .
